# The Influence of the Associated Inactivated Vaccine Against Infectious Rhinotracheitis and Bovine Viral Diarrhea on the Formation and Duration of Colostral Immunity in Kazakh Whiteheaded Calves

**DOI:** 10.3390/vaccines13040408

**Published:** 2025-04-15

**Authors:** Yerbol Bulatov, Alina Kurmasheva, Zhanat Amanova, Ruslan Abitaev, Zhanna Sametova, Asselya Kyrgyzbayeva, Zhanat Kondybaeva, Sholpan Turyskeldi, Abdurakhman Ussembay, Dariya Toktyrova, Dana Mazbayeva, Yeraly Shayakhmetov, Aslan Kerimbayev, Damir Khussainov, Ma Wentao, Aralbek Rsaliyev, Yergali Abduraimov

**Affiliations:** 1 Research Institute for Biological Safety Problems, National Holding “QazBioPharm”, Guardeyskiy 080409, Kazakhstan; zh.amanova@biosafety.kz (Z.A.); r.abitaev@biosafety.kz (R.A.); zh.sametova@biosafety.kz (Z.S.); asselya.91@icloud.com (A.K.); zh.kondybaeva@biosafety.kz (Z.K.); sh.smankizi@biosafety.kz (S.T.); a.ussenbay@biosafety.kz (A.U.); d.toktirova@biosafety.kz (D.T.); d.mazbayeva@biosafety.kz (D.M.);; 2 Department of Veterinary and Zooengineering, Kazakh National Agrarian Research University, Almaty 050010, Kazakhstan; 3 College of Veterinary Medicine, Northwest Agriculture and Forestry University, Xianyang 712100, China; 4 National Holding “QazBioPharm”, Astana 010000, Kazakhstan; a.rsaliyev@qbp-holding.kz (A.R.);

**Keywords:** infectious bovine rhinotracheitis, bovine viral diarrhea, pregnant cows, colostral immunity

## Abstract

**Objectives**: This article presents a study evaluating the antibody levels against infectious bovine rhinotracheitis (IBR) and bovine viral diarrhea (BVD) in Kazakh Whiteheaded calves born to dams immunized with an experimental inactivated combined vaccine against these infections. The vaccine formulation includes the strains “R-93” (IBR) and “Oregon C_24_V” (BVD), which are preserved in the microorganism collection of the Research Institute for Biological Safety Problems. **Methods**: To assess the immune response in newborn calves, blood serum samples were collected before the first intake of colostrum, followed by weekly sampling for 28 weeks post-birth. The antibody response was determined using a virus neutralization assay on MDBK cell cultures and lamb testicle cell cultures. **Results**: The results demonstrated that the protective antibody level against the IBR virus (≥2 log_2_) persisted for up to 25 weeks, while the protective level against the BVD virus (≥3 log_2_) remained for 23 weeks. Based on these findings, the vaccine was deemed safe, as it did not induce abortions or clinical manifestations of the diseases. The overall duration of the colostral immunity in calves against the IBR and BVD viruses reached 23 weeks. **Conclusions**: Therefore, it is recommended that Kazakh Whiteheaded calves be vaccinated with the associated inactivated vaccine against infectious bovine rhinotracheitis and bovine viral diarrhea no earlier than 23 weeks of age.

## 1. Introduction

Infectious bovine rhinotracheitis (IBR) and bovine viral diarrhea (BVD) are infectious diseases characterized by respiratory tract lesions and a detrimental impact on animal health and productivity. The clinical signs of IBR and BVD infections have been well described in the literature [1,2]. These infections are widespread globally, including in the Republic of Kazakhstan. According to serological studies conducted between 2021 and 2022 in four regions of the country (Akmola, Kostanay, Pavlodar, and North Kazakhstan), the overall proportion of IBR-seropositive animals exceeded 45% [3].

In a 2024 monitoring study on BVD conducted in small farms in the Almaty, Mangystau, Karaganda, and Turkistan regions, more than 90% of the samples tested seropositive for the infection [4]. To prevent these diseases, various immunobiological preparations have been developed, including live, inactivated, mono- and polyvalent vaccines. Currently, seven imported vaccines for IBR and BVD prevention are registered in Kazakhstan: Vac-Sules Premium, Bovi-Shield Gold FP5 L5, Inforce 3, Bovimun-4, Vac-Sules IBR, BioBos Respi-4, and BioBos IBR Marker [5].

However, for effective disease prevention, it is essential that the vaccine contains a locally circulating viral strain. In this regard, the Research Institute for Biological Safety Problems has developed an inactivated vaccine, which had demonstrated safety in 6–8-month-old calves.

The primary objective of this study was to investigate the duration of the colostral immunity in calves. We hypothesized that the developed associated inactivated vaccine against IBR and BVD would ensure long-term passive immunity in calves of the Kazakh Whiteheaded breed.

## 2. Materials and Methods

### 2.1. Vaccine and Strains

The primary components of the inactivated combined vaccine are strain “R-93” (IBR) [6] and strain “Oregon C24V” (BVD) [7], both of which are preserved in the “Microorganism Collection” of the Research Institute for Biological Safety Problems. For culturing the IBR virus, Madin–Darby bovine kidney (MDBK) cell cultures were used, which were obtained from the American Type Culture Collection (CCL-22™) [8]. The cell culture was grown at 37 °C under 5% CO_2_ conditions in DMEM growth medium supplemented with 10% fetal bovine serum (Capricorn Scientific, Westport, Connecticut, USA). For culturing the BVD virus, the primary lamb testicle (LT) cell cultures were used, which were provided by the “Laboratory for Cell Biotechnology” of the Research Institute for Biological Safety Problems. The cells were grown as described in a prior article [9]. The cultivation conditions for both viruses were identical: the infectious dose for infecting cells was 0.001 TCID/cell. The infected cells were cultured at 37 °C under 5% CO_2_ in a maintenance medium with fetal bovine serum at a final concentration of 2% and 6% glutamine at a final concentration of 1%. The cultivation periods for the IBR and BVD viruses were 32–36 h. Upon completion of the cultivation, the virus-containing suspensions were subjected to three freeze–thaw cycles, after which they were collected. Before inactivation, the biological activity of the IBR virus was 7.08 ± 0.14 TCID_50_/mL, and for BVD, it was 5.66 ± 0.14 TCID_50_/mL. The virus-containing suspensions were inactivated using formalin. The vaccine was prepared with the Montanide ISA-70 adjuvant (Seppic, Colombes, France).

### 2.2. Animals

This study involved Kazakh Whiteheaded cows, a beef breed whose main characteristics are described in [10]. The selection of animals was based on the following criteria: they had no prior vaccination against IBR and BVD, exhibited no clinical signs of infection, and were seronegative for the IBR and BVD viruses. Water and feed were provided ad libitum throughout the experiment. Initially, the safety of the vaccine was tested on calves aged 6–8 months. For this purpose, the vaccine was administered intramuscularly at a dose twice the immunizing dose, in accordance with the WOAH Manual of Diagnostic Tests and Vaccines for Terrestrial Animals [11]. After confirming the harmlessness, the experiments were continued on pregnant cows. For this, the cows were naturally inseminated, and seven pregnant cows with a gestational age difference of no more than one week were included in this study. The research was approved by the Bioethics Committee of the Research Institute for Biological Safety Problems (approval number 1-14-07-2023). To ensure free access to colostrum, the newborn calves were kept with their dams.

### 2.3. Vaccination of Pregnant Cows

**Figure 1:**
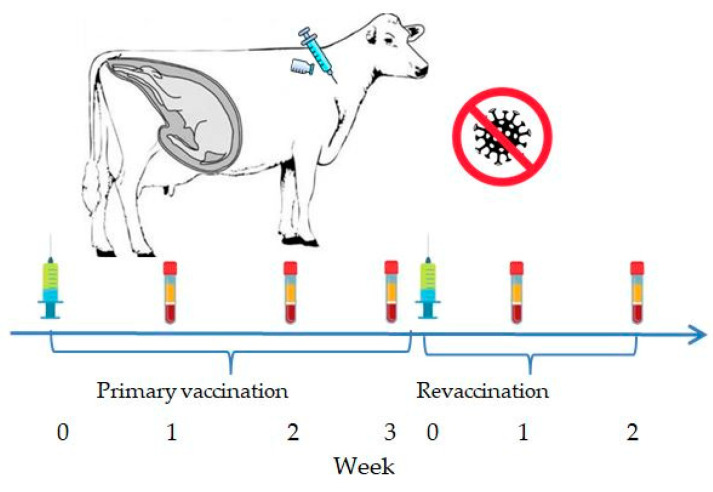
Vaccination and blood sampling scheme for pregnant cows.

In the summer of 2024, four cows in their third trimester of pregnancy were vaccinated intramuscularly in the middle third of the neck with the experimental inactivated combined vaccine against IBR and BVD at a dose of 2 mL (Figure 1).

A booster vaccination was administered three weeks later. The animals were monitored daily, with records kept on their general condition (temperature and any signs of discomfort or disease, including abortion). Blood samples were collected from the jugular vein on weeks 1, 2, and 3 post-primary vaccination and on weeks 1 and 2 post-booster vaccination using vacuum tubes containing a clot activator and gel. Three pregnant cows served as a control group and received no treatment.

### 2.4. Colostral Immunity in Calves

**Figure 2:**
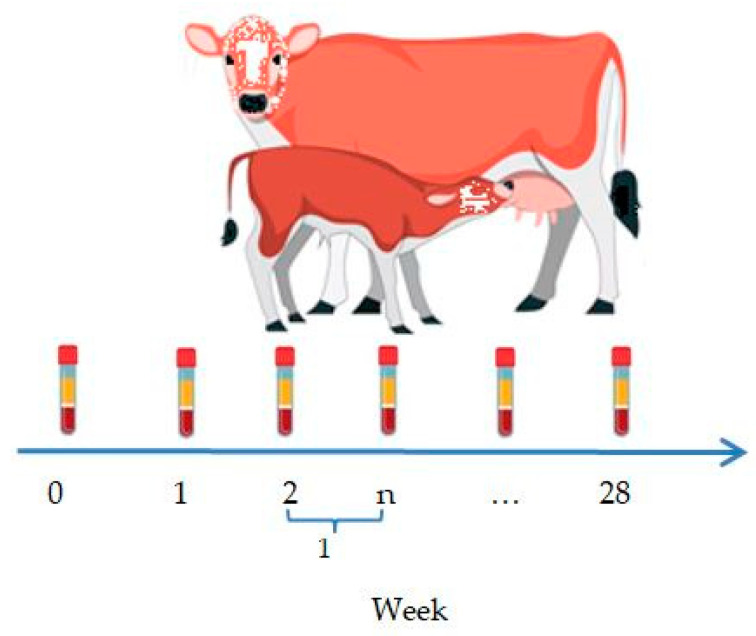
Blood sampling scheme for newborn calves.

Blood serum from the newborn calves was collected immediately after birth, prior to colostrum intake, to detect antibodies against the IBR and BVD viruses. In the calves born to vaccinated cows, the blood sampling continued for 28 weeks at weekly intervals (Figure 2).

### 2.5. Serum Preparation for Serological Assays

For the serological assays, the collected blood samples were incubated at 37 °C for 1 h. The samples were then stored at 2–8 °C according to the MacMillan methodology [12] until complete serum separation. To optimize the serum separation from the coagulated blood, the samples were centrifuged at 3000 rpm for 15 min. The obtained serum samples were stored at −20 °C until further use.

### 2.6. Serological Studies

To determine the immune status of the animals and detect antibodies against the IBR and BVD viruses, virus neutralization assays were performed in triplicate. The methods were conducted according to Raizman et al. [13]. The serum samples were pre-inactivated in a water bath at 56 °C for 30 min. The IBR virus (at a dose of 100 TCID_50_) was incubated with diluted serum samples in 96-well plates on Madin–Darby bovine kidney (MDBK) cell cultures. Similarly, the BVD virus was cultivated on lamb testicle (LT) cell cultures.

### 2.7. Challenge Experiment in Calves

The experiment included the following groups: one calf that acquired antibodies against the IBR and BVD viruses through colostrum from a vaccinated cow, and one calf without antibodies against the target viruses. Both were challenged with the “Oregon C_24_V” BVD strain at 23 weeks of age via intranasal inoculation of 3 mL of virus-containing suspension. Similarly, an equivalent number of calves were infected with the “Colorado-1” IBR strain (purchased from the American Type Culture Collection VR-864™) [14]. The choice of age for the infection was based on the observation that the antibody titers were approaching the minimal protective threshold at that stage. One calf was maintained as a control, receiving no vaccination or viral challenge.

The general health of the animals was assessed (conjunctivitis, nasal discharge, appetite), and blood samples were collected for biochemical analysis, including the total and direct bilirubin, alanine aminotransferase (ALT), aspartate aminotransferase (AST), total protein, and glucose levels. An A-25 BioSystems automatic biochemical analyzer (BioSystems S.A., Barcelona, Spain) was used with the corresponding reagents and consumables.

### 2.8. Statistical Analysis

For the statistical analysis, the serological data were processed using GraphPad Prism software (version 8.0.1). The antibody titers were logarithmically transformed (base 10). The protective neutralizing antibody titers (VNA) were defined as ≥2 log_2_ for IBR and ≥3 log_2_ for BVD [15]. Differences between the antibody titers were determined using a two-way analysis of variance (ANOVA), with statistical significance set at *p* < 0.05.

## 3. Results

When studying the safety of the vaccine in 6–8-month-old calves, no local reactions at the injection site were observed, and the animals remained clinically healthy for the 2-week observation period. In this regard, further studies were conducted on pregnant cows with further study of the colostral immunity of the calves, the results of which are presented in the following paragraphs.

### 3.1. Antibodies to the IBR and BVD Viruses in Pregnant Cows

**Figure 3:**
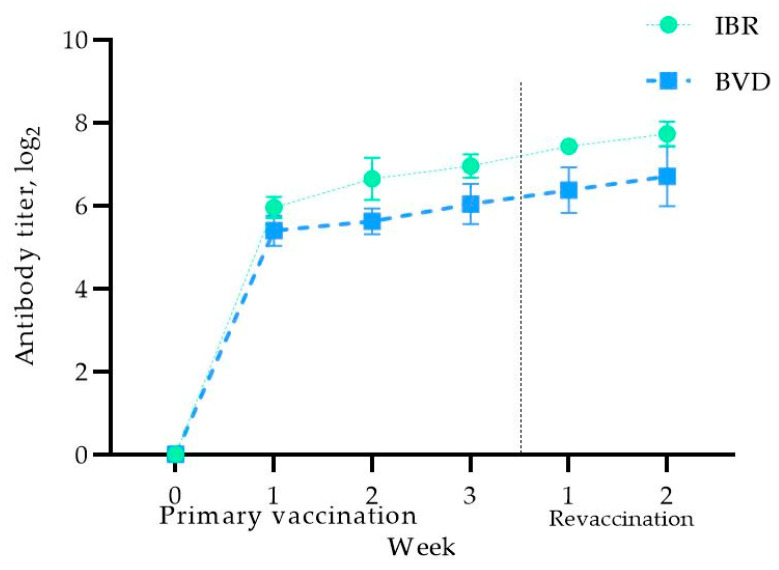
Antibody titer levels in vaccinated pregnant cows.

Before vaccination, no antibodies against the IBR and BVD viruses were detected in the blood serum of the pregnant cows, confirming their seronegative status for the studied infections (Figure 3).

As shown in Figure 3, one week after immunization, the virus-neutralizing antibody (VNA) titers against IBR increased to 5.95 ± 0.25 log_2_, reaching 6.95 ± 0.28 log_2_ at the end of the second week after booster vaccination. The cows remained within physiological norms, with no complications or abortions. A further increase in the antibody titers was observed two weeks after the booster vaccination, reaching 7.72 ± 0.29 log_2_.

Regarding the VNA titers for BVD, a similar trend was observed, with immunity developing in the first week, reaching 5.39 ± 0.36 log_2_, and increasing further to 6.70 ± 0.72 log_2_ by the second week after the booster vaccination. These results indicate that primary vaccination of cows in the third trimester induces adequate antibody titers against the studied infections, which are further enhanced after revaccination, thereby providing protection against IBR and BVD during pregnancy. It is worth noting that the body temperature of the vaccinated cows was within the physiological norm, and therefore the data are not presented in this article.

### 3.2. Antibodies to the IBR and BVD Viruses in Calves with Maternal Antibodies

**Figure 4:**
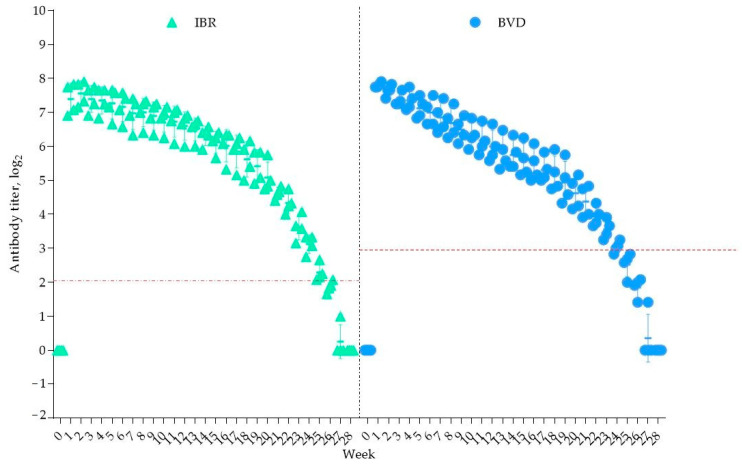
Duration of colostral immunity. The red dashed line indicates the minimum protective antibody titer for the IBR and BVD viruses.

All seven calves were delivered successfully. Before colostrum intake, no antibodies against the studied viruses were detected in the newborns. The results of the blood serum analysis from the four calves that acquired antibodies to IBR and BVD through colostrum from vaccinated cows are presented in Figure 4.

As shown in Figure 4, one week after colostrum consumption, the antibody titers increased to 7.39 ± 0.46 log_2_ for IBR and 7.81 ± 0.07 log_2_ for BVD. A decline in the IBR antibody titers was observed from week 25, falling below the protective level (<2 log_2_) by week 28, at which point antibodies were no longer detectable.

For BVD immunity, the overall duration was 23 weeks, with the antibody titers dropping below the protective threshold (<3 log_2_) from week 24. It is important to note that the decline in the antibody titers for both viruses over the observation period was statistically insignificant (*p* > 0.05). Thus, colostral immunity to IBR persisted for 25 weeks, while BVD immunity lasted for 23 weeks, ensuring protection for the calves for at least five months.

### 3.3. Biochemical Blood Analysis of Calves After Challenge with the IBR and BVD Viruses

No clinical signs of disease (conjunctivitis, nasal discharge) were observed, and the animals’ appetite did not decrease. The biochemical blood analysis provided an overall assessment of the animals’ health, revealing potential deviations related to the viral infections (Table 1).

As shown in Table 1, all the infected calves exhibited a decrease in their glucose levels (<2.2 mmol/L) compared to the control calf (3.3 mmol/L). In the unvaccinated calf infected with IBR, the total bilirubin levels increased to 20.5 mg/dL, while the total protein levels decreased to 68.2 g/L. Similarly, in the unvaccinated calf infected with BVD, the total bilirubin increased to 40 mg/dL, and the total protein dropped to 60.3 g/L.

In contrast, the biochemical parameters of the control calf remained within acceptable limits. These differences between the vaccinated and unvaccinated calves can be attributed to their differing immune statuses, as the antibodies induced by vaccination provided better protection against the infections.

## 4. Discussion

One of the stressful periods in a cow’s life is calving, while for a newborn calf, it is the time before colostrum intake, as both are at a high risk of infection during this period [17]. Compared to other animals, bovine fetuses exhibit agammaglobulinemia, except in cases of intrauterine infection, meaning that antibodies in cattle do not cross the placental barrier [18]. One of the reasons for the persistence of diseases such as bovine viral diarrhea (BVD) in herds is the birth of persistently infected calves that were infected in utero [19]. Consequently, newborn calves are highly dependent on maternal antibodies, which are passively transferred through colostrum [20]. As the results of the work [21] showed, the VNA titers against IBR in vaccinated pregnant cows were increased in comparison with the control ones, which subsequently allowed the transfer of passive immunity against the infection to the calves through colostrum. Colostrum contains a high concentration of antibodies, and its protective effect usually lasts for at least one month [22]. According to research [23], newborn calves on day 0, before colostrum intake, have low serum maternal antibody concentrations; however, these levels significantly increase within 24 h. Notably, early vaccination of calves may not provide satisfactory immunity, as antibodies generated by passive immunity can neutralize viral particles contained in the vaccine [2]. At the same time, colostrum from immunized dams provides passive immunity to calves, which can persist until 4–6 months of age for infectious bovine rhinotracheitis (IBR) virus [24] and 5–9 months for BVD virus [25]. Therefore, vaccinating pregnant cows increases the chances of passive immunization of the offspring. The veterinary market offers a wide range of immunobiological prophylactic products containing attenuated, inactivated, and modified viruses for the prevention of IBR and BVD.

The authors of [26] observed a slow decline in maternal antibody levels against BVD in calves whose dams had high antibody titers following vaccination before calving. A study [27] examined two groups of experimentally infected calves: one group received colostrum containing anti-BVD antibodies, while the other consumed colostrum without such antibodies. The results indicated that the first group exhibited no clinical signs of disease, whereas the second group was not protected from infection. Vaccination of pregnant beef cows with a modified live vaccine against IBR and BVD enabled maternal immunity to provide an adequate level of protection for calves exposed to infected cattle [28]. Furthermore, intramuscular vaccination of pregnant Brahman cows with a modified vaccine resulted in high antibody titers against the IBR and BVD viruses by the second week after administration of the immunobiological preparation [29].

Vaccines containing inactivated viruses are considered a safer option for pregnant cows. Preventive measures against IBR and BVD using these vaccines have not resulted in adverse reactions in cows prior to calving, and the antibody titers against these infections were detected in both serum and colostrum [30].

Adjuvants play a crucial role in inactivated vaccines by ensuring a strong immune response. Research in guinea pigs demonstrated that the oil-based adjuvant ISA-61 in a seven-component vaccine against IBR and BVD was significantly more effective than ISA-50, as it promoted the production of higher antibody levels [31]. In our studies in pregnant cows, a vaccine containing the ISA-70 adjuvant demonstrated both immunogenicity and safety. Therefore, selecting an appropriate adjuvant is a critical step in vaccine formulation, as different adjuvants can have varying effects on the vaccinated organism.

Studies have been conducted to assess the impact of seasonal vaccination (in summer and autumn) on the development of antibodies against IBR and BVD, with no significant differences observed in the results [32]. However, these studies were conducted in Pakistan, where the winters are relatively warm [33], whereas in Kazakhstan, the temperatures in the southern regions range from −18 °C to −28 °C [34]. Our studies on the vaccination of pregnant cows were conducted in summer; thus, the impact of autumn vaccination remains uncertain. This aspect can be explored in future research to expand knowledge on the developed immunopreparation.

In a study [35], pregnant cows were immunized with a commercial Argentine vaccine containing inactivated IBR and BVD strains. The results revealed that newborn calves developed colostral immunity against IBR; however, no antibodies against BVD were transmitted through colostrum. This could be explained by the fact that BVD-infected bovine fetuses become immunologically tolerant to the infection and, as a result, remain persistently infected and never develop immunity to it [36]. The findings of another study [37] demonstrated that calves responded effectively to the IBR and BVD vaccines only after passive immunity had waned.

Research [38] showed that 62% of blood serum samples from 3-month-old calves born to cows vaccinated against IBR had undetectable antibody levels, while the remaining samples exhibited antibody levels at the lowest detectable thresholds. Our studies revealed the presence of colostral immunity against IBR and BVD in calves within one week after consuming colostrum from vaccinated dams. The duration of the passive immunity against both viruses lasted for at least 23 weeks.

According to study [15], a virus-neutralizing antibody (VNA) titer of ≥2 log_2_ is considered protective against IBR infection, while a VNA titer of ≥3 log_2_ is required for BVD protection. Additionally, study [39] demonstrated that calves with VNA titers < 4 log_2_ against BVD developed severe clinical disease following experimental infection. In our study, control infections of calves, including animals with antibody titers ≥ 3 log_2_ and without antibodies for both viruses before challenge, were used. Unfortunately, the titers of VNA in the calves after challenge were not measured in the current study. We acknowledge this as a limitation and will consider including such data in future work. It is worth adding that no clinical signs characteristic of either IBR or BVD, including fever, nasal discharge, and respiratory symptoms, were observed in any of the animals from either the control or vaccinated groups. In the current study, calves were used to investigate the biochemical changes in the blood. Deviations in the protein levels from the norm indicate metabolic disturbances in the organism [40]. According to study [41], the total protein levels in IBR-infected cows remained unchanged, similar to those in healthy cattle. Likewise, in BVD-infected cows, the total protein levels did not significantly differ from those of healthy animals [42]. In our studies, unvaccinated calves exhibited a decrease in the total protein levels after infection, whereas vaccinated and control calves maintained values within physiological norms.

In the serum of IBR-infected animals, the glucose levels were significantly elevated [41]. At the same time, studies on postpartum metritis in cows, which may be associated with BVD, showed that infected animals exhibited hypoglycemia (low blood glucose levels) compared to healthy control animals [43]. A decrease in the blood sugar levels is indicative of depleted glycogen reserves in the liver and muscles [40]. In our study, all the calves subjected to viral infection demonstrated a reduction in the glucose levels, whereas the control calf maintained values within the normal range.

The determination of the bilirubin levels in the blood provides insight into the liver’s ability to secrete bile [40]. In a study in cows with nodular dermatitis, which shares certain clinical similarities with BVD, the levels of total and direct bilirubin were significantly elevated in infected animals, indicating liver dysfunction [44]. These findings are relevant to BVD, as the virus can cause liver damage and oxidative stress, leading to increased bilirubin levels [45]. In contrast, the total and direct bilirubin levels remained stable in IBR-infected animals [41]. In our study, the total bilirubin levels in all the calves were within acceptable limits; however, the direct bilirubin levels were elevated in unvaccinated, infected calves.

In biochemical analysis, ALT and AST serve as indicators of cellular damage in internal organs [40]. A significant increase in these enzymes is a marker of pronounced cytolysis and damage to liver or muscle tissue. In IBR infection, a substantial elevation of ALT and AST was observed in a previous study [41]. However, in another study [42], the ALT levels in BVD-infected cows were not significantly affected, suggesting that virus-induced liver damage does not always lead to increased enzyme levels. Conversely, the AST levels were notably higher in infected animals compared to healthy controls. In our study, these enzyme levels remained within the normal range, with no deviations observed.

Although biochemical markers provide valuable information about animal health, it is essential to consider that each organism is unique. Therefore, study results may be influenced by numerous factors, including genetic traits and even seasonal environmental changes [1]. Our study was conducted in Kazakh Whiteheaded beef cattle, which were in the third trimester of pregnancy during the summer and were vaccinated accordingly. The calves were born in early autumn, and the level of colostral immunity undoubtedly depended on the health status of their mothers as well as the environmental conditions.

The results of our study demonstrated that the experimental inactivated associated vaccine against IBR and BVD is safe for pregnant cows, as it did not exert any adverse effects during gestation. Furthermore, immunity was established within one week after primary vaccination. In turn, newborn calves from healthy, vaccinated mothers had no detectable antibodies to these infections before consuming colostrum; however, antibodies appeared within the first week after colostrum intake. This suggests that vaccination of pregnant cows elicited an antibody response sufficient to be transferred to their offspring through colostrum. The duration of the colostral immunity in our calves lasted for at least 23 weeks. Our hypothesis that the developed vaccine would be able to provide long-term passive immunity in calves of the Kazakh Whiteheaded breed turned out to be correct. Upon challenge infection, the biochemical profile of the vaccinated calves was significantly better compared to the unvaccinated ones.

Nevertheless, vaccine testing cannot fully replicate complex field conditions, which may involve multiple simultaneous infections, including bacterial pathogens. Moreover, not all diseases present with identical symptoms, and the presence of an infection does not always lead to alterations in all biomarkers. Therefore, it is crucial to use these markers in combination with other diagnostic methods and ongoing health monitoring of the animals. In this regard, further research should be conducted on commercial farms to assess the effectiveness of the inactivated associated vaccine against IBR and BVD and to determine whether the induced immunity provides reliable protection against these infections. In the near future, after registration trials using a large number of livestock, we may be analyzing the well-being of farms in terms of both infections in comparison with farms with unvaccinated animals.

## 5. Conclusions

This study demonstrated that the use of the associated inactivated vaccine against IBR and BVD has a lasting impact on the immune status of Kazakh Whiteheaded calves. Calves born to dams vaccinated with this vaccine maintained a protective antibody level against the IBR virus for up to 25 weeks, and against the BVD virus for up to 23 weeks. The overall duration of the colostral immunity lasted for 23 weeks. Based on these findings, it is recommended that Kazakh Whiteheaded calves be vaccinated with the vaccine no earlier than 23 weeks of age, ensuring reliable and long-lasting immune protection. This conclusion emphasizes the importance of vaccination in preventing infectious diseases and maintaining the health of livestock.

## Figures and Tables

**Table 1:** Biochemical blood parameters of calves infected with IBR and BVD.

№	Calf Status	Biochemical Parameter	Permissible Concentration [16]	Concentration on Day 7 After Infection	Units of Measurement
1	V, IBR	ALT	1.3–60	26.6	U/L
		AST	11–160	30.6	U/L
		Bilirubin direct	0.19–17	0.25	mg/dL
		Bilirubin total	0.7–14	13.8	mg/dL
		Glucose	2.2–4.4	1.3 *	mmol/L
		Protein total	72–90	72.2	g/L
2	NV, IBR	ALT	1.3–60	13.2	U/L
		AST	11–160	33.9	U/L
		Bilirubin direct	0.19–17	0.60	mg/dL
		Bilirubin total	0.7–14	20.5 *	mg/dL
		Glucose	2.2–4.4	1.3 *	mmol/L
		Protein total	72–90	68.2 *	g/L
3	V, BVD	ALT	1.3–60	11.6	U/L
		AST	11–160	31.7	U/L
		Bilirubin direct	0.19–17	0.28	mg/dL
		Bilirubin total	0.7–14	8.1	mg/dL
		Glucose	2.2–4.4	1.2 *	mmol/L
		Protein total	72–90	72.2	g/L
4	NV, BVD	ALT	1.3–60	19.9	U/L
		AST	11–160	38.9	U/L
		Bilirubin direct	0.19–17	0.31	mg/dL
		Bilirubin total	0.7–14	40 *	mg/dL
		Glucose	2.2–4.4	1.2 *	mmol/L
		Protein total	72–90	60.3 *	g/L
5	Control, NV, WI	ALT	1.3–60	29.7	U/L
		AST	11–160	36.0	U/L
		Bilirubin direct	0.19–17	0.36	mg/dL
		Bilirubin total	0.7–14	2.2	mg/dL
		Glucose	2.2–4.4	3.3	mmol/L
		Protein total	72–90	76.6	g/L

* deviation from the norm; V, vaccinated; NV, not vaccinated; IBR, challenge with IBR virus; BVD, challenge with BVD virus; WI, without infection.

## Data Availability

Data are contained within the article.

## Abbreviations

The following abbreviations are used in this manuscript:

IBR	Infectious bovine rhinotracheitis
BVD	Bovine viral diarrhea
RK	Republic of Kazakhstan
VNA	Virus-neutralizing antibodies
AST	Aspartate aminotransferase
ALT	Alanine aminotransferase
